# Towards all-oral and shorter treatment regimens for drug-resistant tuberculosis

**DOI:** 10.2471/BLT.18.223230

**Published:** 2018-10-01

**Authors:** Karin Weyer, Dennis Falzon, Ernesto Jaramillo

**Affiliations:** a Global Tuberculosis Programme, World Health Organization, 1211 Geneva 27, Switzerland.

The treatment of drug-resistant tuberculosis has attracted intense debate over many years because of the limited number of available drugs and the uncertainties about the relative effectiveness of regimens used, especially those to treat rifampicin- or multidrug-resistant (MDR) tuberculosis. Many treatment options fail to achieve a lasting cure in a large proportion of patients with MDR tuberculosis and have a very unfavourable safety profile.^1^ Injectable agents requiring daily, painful, intramuscular administration for several months have been particularly problematic, at times leading to irreversible deafness and other harms.

Given the need to deliver life-saving treatment when choice of medicines is limited, patients and clinicians have had to work with toxicity of MDR tuberculosis regimens. Due to a lack of alternative medicines, treatment guidance from the World Health Organization (WHO), the American Thoracic Society, the United States Centers for Disease Control and Prevention and others, has historically featured injectable agents (amikacin, kanamycin and capreomycin) among priority medicines for MDR tuberculosis regimens, largely based on limited available evidence and expert clinical opinion.

Since 2011, WHO guidelines for drug-resistant tuberculosis have been developed using the Grading of Recommendations Assessment, Development and Evaluation evidence-based approach.^2^ This widely-used system improves transparency in public health policy development, indicating which evidence is considered relevant, how systematic reviews and data analyses are done, how quality of evidence is judged and how conflict of interest is managed. Policy recommendations using this approach include conditions for their implementation and monitoring. One such measure for WHO MDR tuberculosis treatment policies is active tuberculosis drug safety monitoring and management, motivated by the increasing roll-out of newer medicines or novel regimens for which there is limited experience.^3^

Despite appeals by patients and carers for more effective and safer regimens, evidence-based patient care and public health policy development for MDR tuberculosis have been negatively impacted by the long-standing gaps in research investment.^4^ Randomized controlled trials for MDR tuberculosis are complex and expensive to conduct, taking several years to complete. Recent trials have provided limited evidence for the replacement of injectable agents and other more toxic regimen components with newer, safer options. Delamanid, one of the two tuberculosis agents released in recent years, did not influence final cure in its Phase III trial when used in addition to injectable-containing longer MDR tuberculosis regimens.^5^ Available trial evidence for bedaquiline comes from to a Phase IIb study in which it was also accompanied by injectable agents.^6^ The sparse evidence reported from trials of clofazimine and linezolid, other agents used in MDR tuberculosis regimens, also did not define their role in replacing injectable agents.

This situation is, however, expected to improve as more evidence becomes available for safer and more effective treatment, newer medicines become accessible to more patients and monitoring and rapid management of drug adverse effects are intensified. WHO has repeatedly stressed the need to balance effectiveness and harms when choosing medicines in MDR tuberculosis regimens. Options to replace injectable agents when serious adverse events occur have been highlighted in WHO guidance.^7^ Moreover, streptomycin-containing regimens are no longer recommended in drug-susceptible tuberculosis and WHO now also recommends a six-month injectable-free treatment for most cases of isoniazid-resistant tuberculosis.^8^^,^^9^ Since 2016, WHO recommends that injectable agents may be avoided in MDR tuberculosis regimens for children with mild disease.^10^ Furthermore, in August 2018, WHO announced that its forthcoming MDR tuberculosis treatment guidelines will favour an all-oral regimen for most patients.^11^

The landscape of MDR tuberculosis treatment has changed dramatically in the last 10 years. Among others, evidence for the composition and duration of regimens has evolved; new oral medicines have become accessible (facilitated in part by mechanisms such as the bedaquiline donation programme coordinated by the United States Agency for International Development); significant price reductions for key medicines have been achieved through the Global Drug Facility; access to molecular and phenotypic drug susceptibility testing has expanded; and better quality data with a global span on effectiveness and safety from ongoing trials, observational cohort studies and programmatic use of second-line tuberculosis medicines are increasingly available. New data from MDR tuberculosis patients treated with all-oral regimens lasting nine months or less are expected in 2019.

The era when patients with drug-resistant tuberculosis had to endure injectable agents as a necessary evil to achieve a durable cure is ending. Accelerated innovation and increased investment in research should translate into a more personalized and tolerable pathway to cure for many more patients with drug-resistant tuberculosis.
